# Improving physical health care for inpatients with eating disorders

**DOI:** 10.1192/bjo.2021.539

**Published:** 2021-06-18

**Authors:** Edward Knights, Monique Schelhase, Rhys Jones, Lou Burke

**Affiliations:** LYPFT NHSFT

## Abstract

**Aims:**

Primary aim – To improve how physical health issues are addressed for inpatients with eating disorders

Secondary aim – To improve efficiency within the MDT

**Background:**

The Yorkshire Centre for Eating Disorders (YCED) is an inpatient unit for the treatment of patients with anorexia and bulimia nervosa. Anorexia nervosa has the highest mortality of all psychiatric disorders with an extensive list of physical manifestations. This project was designed to help better address the physical health concerns of our patients by introducing a primary care style, once weekly clinic that patients could self-refer to.

**Method:**

Questionnaires were designed to assess whether a once weekly physical health clinic would benefit the service.

The clinic was run on a weekly basis from 26th April to 24th June 2019. Follow-up questionnaires were designed and distributed to both patients and staff following this period. Data were analysed with Microsoft Excel to determine if improvement had been made.

**Result:**

N = 12 inpatients responded to the initial questionnaires, n = 2 were discharged during the 8 week period so were included in the analysis but did not complete the follow-up questionnaire.

100% of the staff (n = 8) felt a once weekly clinic would benefit their patients. 62% (n = 5) stated they felt distracted from their other duties with physical health requests.

33% (n = 4) of the inpatient group felt the clinic would benefit them with 67% (n = 8) stating indifference to the idea.

26 appointments were conducted in the physical health clinic with 80% (n = 8) of the service users accessing at least once. 70% (n = 7) stated their physical health concerns had been better addressed since the clinic had been started.

90% (n = 9) of inpatients and 90% (n = 9) of staff responded that the physical health clinic should remain permanent. 90% (n = 9) of staff stated they had more time for their other duties since the introduction of the clinic.

Prior to the clinic 63% (n = 5) of staff responded that in a typical day they were approached between 2-5 times for physical health requests with the other 37% (n = 3) being approached once.

Following the clinic 80% (n = 8) of staff responded that they were approached once in a typical working day.

**Conclusion:**

The qualitative data from the questionnaires indicated success in both improving patient care and reducing nursing workload.

The physical health clinic has been made a permanent feature on the ward and has been continued by the incoming foundation doctor and ward ANP.

